# Functional Classification of the ATM Variant c.7157C>A and *In Vitro* Effects of Dexamethasone

**DOI:** 10.3389/fgene.2021.759467

**Published:** 2021-10-25

**Authors:** Sara Biagiotti, Ambra Barone, Mattia Paolo Aliano, Giulia Federici, Marco Malatesta, Caterina Caputi, Silvia Soddu, Vincenzo Leuzzi, Luciana Chessa, Mauro Magnani

**Affiliations:** ^1^ Department of Biomolecular Sciences, University of Urbino, Urbino, Italy; ^2^ Department of Research and Advanced Technologies, IRCCS Regina Elena National Cancer Institute, Roma, Italy; ^3^ Department of Chemistry, Life Sciences and Environmental Sustainability, University of Parma, Parma, Italy; ^4^ Department of Human Neuroscience, Sapienza University of Rome, Roma, Italy; ^5^ Sapienza University of Rome Foundation, Roma, Italy

**Keywords:** ataxia telangiectasia, ataxia telangiectasia-mutated, ataxia telangiectasia-mutated variants, dexametasone, variant with uncertain significance, phenotype

## Abstract

Most of the ATM variants associated with Ataxia Telangiectasia are still classified as variants with uncertain significance. Ataxia Telangiectasia is a multisystemic disorder characterized by “typical” and “atypical” phenotypes, with early-onset and severe symptoms or with late-onset and mild symptoms, respectively. Here we classified the c.7157C > A ATM variant found in homozygosity in two brothers of Lebanese ethnicity. The brothers presented with an atypical phenotype, showing less than 50% of the positive criteria considered for classification. We performed several in silico analyses to predict the effect of c.7157C > A at the DNA, mRNA and protein levels, revealing that the alteration causes a missense substitution in a highly conserved alpha helix in the FAT domain. 3D structural analyses suggested that the variant might be pathogenic due to either loss of activity or to a structural damage affecting protein stability. Our subsequent *in vitro* studies showed that the second hypothesis is the most likely, as indicated by the reduced protein abundance found in the cells carrying the variant. Moreover, two different functional assays showed that the mutant protein partially retains its kinase activity. Finally, we investigated the *in vitro* effect of Dexamethasone showing that the drug is able to increase both protein abundance and activity. In conclusion, our results suggest that the c.7157C > A variant is pathogenic, although it causes an atypical phenotype, and that dexamethasone could be therapeutically effective on this and possibly other missense ATM variants.

## Introduction

Ataxia Telangiectasia (AT) is a rare autosomal recessive disorder caused by biallelic mutations in the ATM gene (Savitsky et al., 1995). AT is characterized by early and progressive neurodegeneration with cerebellar ataxia, telangiectasia, immunodeficiency (mainly characterized by IgG and IgA deficiency and CD4^+^ lymphocytopenia), growth retardation and sexual immaturity, and strong predisposition to tumours, in particular lymphoma and leukaemia (Boder and Sedgwick, 1958; Rothblum-Oviatt et al., 2016).

The ATM gene codifies for a powerful protein kinase belonging to the PI3-kinase family (Abraham, 2004). Wild-type ATM (WT-ATM) protein is mainly found in two different conformations: “closed dimer,” with low enzymatic activity, and “open dimer,” with high enzymatic activity (Baretic et al., 2017). In response to various stimuli, ATM activation requires phosphorylation of the closed ATM dimers and dissociation into active ATM monomers, so allowing many processes that are lost in AT patients (Shiloh and Ziv, 2013).

AT is a multisystemic pathology characterized by a highly variable phenotype (Gilad et al., 1998). The typical phenotype is caused by nonsense mutations that result in truncated and non-functioning forms of the expressed protein; otherwise, missense mutations and in frame deletions/insertions can lead to the production of mutant ATM proteins that partly preserve their activity (Chun and Gatti, 2004). Based on the type of mutation and the functionality of the resulting protein, ATM variants are classified into benign, mildly pathogenic, pathogenic, or variant with uncertain significance (VUS) when their consequences on the phenotype are not clear. Patients with AT carrying typical ATM variants have a severe clinical picture characterized by early onset ataxia before 8 years, loss of the ability to walk, oculomotor apraxia and ocular telangiectasia before the age of 15, and clinical immunodeficiency with IgA deficiency in 60% of cases (Fievet et al., 2019). On the other hand, AT patients with atypical variants show a less severe phenotype with late-onset ataxia after 8 years, loss of ability to walk after 15 years, and non-manifest immunodeficiency (Fievet et al., 2019).

Thus far, AT is an incurable disease. However, clinical studies have shown that the treatment with glucocorticoids (GC), in particular oral betamethasone (Buoni et al., 2006; Broccoletti et al., 2008; Broccoletti et al., 2011; Quarantelli et al., 2012; Zannolli et al., 2012; Cirillo et al., 2018) and intra-erythrocyte dexamethasone (DEX) (Chessa et al., 2014; Leuzzi et al., 2015), is able to ameliorate neurological symptoms of AT patients. Several studies have been conducted on the mechanism of action of GC in AT, leading to the hypothesis that their beneficial effect is due to a combination of transcription and protein effects which acts on different metabolic and oxidative pathways (Russo et al., 2009; Menotta et al., 2012; Giardino et al., 2013; Biagiotti et al., 2014; Biagiotti et al., 2016; Menotta et al., 2017a; Menotta et al., 2017b; Menotta et al., 2018). However, the effect of DEX on patients with atypical AT or missense mutations has not yet been evaluated.

In this paper, the effect of the drug on cells of two patients of Lebanese ethnicity, double mutants for the missense mutation c.7157C > A (NM_000,051.4) leading to the production of the ATM variant p. Ala2386Glu, not yet classified and not fully described in the literature, was evaluated. First, *in silico* analyses were carried out to assess the effect of the missense mutation on the primary and secondary/tertiary structure of the protein and on its activity. Then, *in vitro* experiments were performed for the identification and quantification of the mRNA and the mutant protein in the lymphoblastoid cell lines (LCLs) derived from the two patients. Finally, two biological tests were carried out to verify protein functionality. Experiments were conducted both in the absence and in the presence of DEX to investigate whether this missense variant is responsive to this treatment and the drug might represent a therapeutic option for those patients with this ATM missense variant.

## Materials and Methods

### Patients and Phenotypic Description

The Lebanese family was identified through analysis of the full-length ATM gene in the older child, who first reported difficulties in gait and posture; a written informed consent was obtained from the father for the use of clinical data and for the use of cell lines already deposited in the cell bank for further investigation. The test revealed a homozygous nucleotide change (c.7157C > A) in exon 49 of the ATM gene; at the time of diagnosis, this change was not yet described but it has been associated with AT only in 2019 (Fievet et al., 2019). The same mutation was found in homozygosity in the younger brother and in simple heterozygosity in the two parents (carriers).

The propositus (AT1-54000) was born from consanguineous parents after a normal pregnancy. Postural instability was noticed during the first years of life but did not prevent autonomous walking. The diagnosis of AT was made at the age of 12. Electrophysiological study showed a sensorimotor polyneuropathy and brain MRI disclosed cerebellar atrophy. From the age of 18, the propositus experienced a progressive worsening of gait, requiring the wheelchair for longer walk. Respiratory infections were not reported. From the immunologic point of view, IgA deficiency and lymphopenia were documented while no lymphoproliferative disorders were observed. Hepatic steatosis revealed by abdominal ultrasound was accompanied by increased plasmatic levels of gamma-GT and triglycerides. At the age of 20 (last visit before the present study), gait was possible only for a few steps because of a remarkable trunk and limb ataxia, generalized rigidity, lower limbs weakness due to peripheral neuropathy and dystonic postures of the upper limbs. Ocular motility was characterized by impairment of saccadic pursuit, increased gain of vestibulo-ocular reflex (VOR), and hypometric and slow saccades (mild oculo-motor apraxia). Scanning but essentially intelligible speech was reported together with a preserved cognitive functioning. From a video sent by the father at the age of 24 (time of the present study) the patient lost the autonomous gait without support.

Like the propositus, his younger brother (AT1-54001) was born from a normal pregnancy but postural instability was reported from an early period. The molecular diagnosis of AT was made at the age of 3, while cerebellar atrophy was detected later by brain MRI. The clinical history was characterized by recurrent episodes of bronchitis for which he received immunoglobulin infusion every 21 days; a B-cell lymphoma was diagnosed at the age of 7. Immunologic parameters revealed lymphopenia and hyper IgM, while the IgA values were fluctuating. At the age of 11 (last visit before the present study), the younger brother showed truncal ataxia, still compatible with autonomous ambulation, dysmetria, action and postural tremor, dysdiadochokinesia and a scanned but still intelligible speech. Oculomotor apraxia and initial signs of peripheral neuropathy were observed. From a video sent by the father at the age of 15 (age of the present study), the boy was still walking independently, although with a rigid type of pattern. As it can be estimated from the video, a worsening of peripheral neuropathy and oculo-motor apraxia were detected. Unfortunately, the younger child died before the ending of this work for the recrudescence of a very aggressive diffuse large B-cell lymphoma.

### *In silico* Analyses

To predict the disease-causing potential of DNA variants, we chose Mutation Taster, a free web-based application developed by Schwarz et al. (Schwarz et al., 2014). To estimate the impact of a variant on the gene product/protein, the software compares integrated databases (e.g., Ensembl, UniProt, ClinVar, ExAC, 1,000 Genomes Project, phyloP, phastCons) to discard known, harmless polymorphisms, then tests the remaining variants according to the type of gene alteration (i.e., silent synonymous or intronic alterations that do not lead to an amino acid exchange, mutations that affect a single amino acid, mutations that cause complex changes in the amino acid sequence). The multiple tests performed by the software comprise amino acid substitution(s), conservation of affected amino acid(s), potential loss of functional protein domains, length of protein, effect on splicing, conservation at DNA level, potential abrogation of regulatory elements (such as transcription factor binding sites), etc. The single results are then assessed by a Naive Bayes classifier, which decides whether their combined effect might be deleterious for the protein.

Additional analyses of possible alternative splice sites were performed by MaxEntScan, SpliceAI and Human Splicing Finder (HSF). In the first case, we used all the possible nine positions surrounding the mutation, as required by MaxEntScan input. In the second one, we used the vcf file deposited in ClinVar FTP (https://ftp.ncbi.nlm.nih.gov/pub/clinvar/vcf_GRCh37/clinvar.vcf.gz) to extract the chr11: 108199815C > A for the GRCh37 assembly. In addition, we used the one-stop-shop algorithms and matrices of the HSF system to identify all splicing signals including acceptor and donor splice sites, branch points, and auxiliary splicing signals (ESE and ESS).

To evaluate the conservation degree of the residue Ala2386, the CLC Sequence Viewer software was used to perform a multiple alignment of FASTA sequence of human ATM with its orthologous proteins from *Mus musculus* (mouse), *Gallus* (chicken), *Xenopus tropicalis* (Western clawed frog), *Danio rerio* (Zebrafish), *Nematostella vectensis* (Starlet sea anemone), *Trichoplax adhaerens* (Trichoplax reptans), *Drosophila melanogaster* (Fruit fly).

Structural analysis of ATM p. Ala2386Glu mutant protein was performed by two different web services: Missense3D, which exploits PHYRE2 Protein Fold Recognition Server, and DynaMut. The obtained structural models were aligned by PyMOL software (Shroedinger, Ind.) with the WT-ATM protein PDB ID 6K9L (Xiao et al., 2019), which represents the closed dimer of ATM and was used as reference structure and template in both modelling software.

### Cell Cultures and Treatments

The LCLs used in this study were 243RM (WT-ATM) and AT129RM (ATM-null), already available in our laboratory and previously described (Menotta et al., 2012), and the two brothers’ lines. The two AT LCLs (AT1-54000 and AT1-54001) derived from the two brothers of Lebanese ethnicity and homozygous for the mutation c.7157C > A were established by Prof. Dominique Stoppa-Lyonnet and kindly purchased by the Genethon Institute of Paris. LCLs were grown in complete RPMI medium (15% heat-inactivated FBS, 1% l-Glutamine, 2% Penicillin/Streptavidin and 1% non-essential amino acids) and kept at 37°C in a humidified incubator with 5% CO_2_. For treatments, cell viability was calculated and if evaluated above 90%, the cells were plated at the concentration of 8 × 10^5^ cells/ml and subjected to stimulation with 100 nM DEX for 24 h. At the end of treatment, cells were collected and processed for mRNA and protein extractions and/or immunofluorescence analyses.

### Western Blot

Cells were centrifuged for 5 min at 1,200 rpm and washed with PBS. Cell pellets were lysed for protein extraction with RIPA buffer (100 mM Tris-HCl/pH 7.5, 0.1 M NaCl, 1 mM EDTA, 1% Triton X-100, 0.5% sodium deoxycholate, 0.1% sodium dodecyl sulphate) completed with a cocktail of protease and phosphatase inhibitors. Protein extracts (20/40 µg) were loaded onto 4–15% Mini-PROTEAN^®^ TGX Stain-Free™ Protein Gels and transferred onto 0.2 µm nitrocellulose filters. The primary antibodies (Abs) used were ATM1B10 (AbCam), *p*-CHK2 (Thr68) (CST # 2661), CHK2 (1C12) (CST # 3440). Subsequently, nitrocellulose filters were incubated with the secondary Abs Goat Anti-Rabbit/Mouse IgG HRP-Conjugated (Biorad) for 1 h at room temperature and chemiluminescence reaction visualized by ChemiDoc MP Imaging system (Biorad). Total protein normalization via stain-free technology was used to normalize Western blot signals (Gurtler et al., 2013).

### Studies at the mRNA Level

Total RNA was extracted from LCLs, treated or not with DEX, using the RNeasyPlus mini kit (Qiagen) and cDNA was obtained by PowerScript reverse transcriptase (Clontech). Quantitative PCR for gene expression were performed by TaqMan Gene Expression Assays using the TaqMan Gene Expression Master Mix (Life Technologies) and run on a 7500 Real-Time PCR System (Applied Biosystems). For native ATM transcript quantification, we used the Hs01112314_m1 assay ID (ThermoFisher Scientific) that spans between exons 48–49; HPRT1 was used as a housekeeping gene.

End-point PCRs were performed on the same cDNA samples to identify any alternative splicing driven by the alteration. Two pairs of specific primers (Supplementary Table S1) were designed to amplify the regions from the start of exon 48 to the end of exon 50 and from the start of exon 48 to the end of exon 63 (last exon, using NM000051.4 as reference sequence).

### CHK2 Activation Assay

ATM kinase activity was assessed based on CHK2-Thr68 phosphorylation as reported in (Fievet et al., 2019) with minor modifications. Briefly, cells were plated and treated with Camptothecin (CPT) for 1 h at 37°C. CPT treatment was performed on 243RM, AT129RM, AT1-54000, and AT1-54001 LCLs, which were previously treated (or not) with DEX. At the end of treatment, cells were collected and subjected to Western blotting.

### Immunofluorescence Analysis

Proliferating LCLs were centrifuged onto poly-l-lysine-coated coverslips, fixed, permeabilised, blocked and stained as described (Prodosmo et al., 2013). The following Abs were employed: mouse monoclonal anti-p53 (DO-7 1:100, DAKO, Santa Clara, CA, United States), rabbit anti γ-Tubulin (1:800, Sigma-Aldrich, St. Louis, MO, United States); 488-conjugated goat anti-rabbit and 594-conjugated goat anti-mouse polyclonal Abs (both 1:400, Alexa-Flour, Invitrogen). Immunofluorescence signals were examined through an Olympus BX53 microscope equipped with epifluorescence and photographs were taken (×100 objective) using a cooled camera device (ProgRes MF). p53-mitotic centrosome localization (p53-MCL) was assessed in >70 mitotic cells/sample as previously described (Prodosmo et al., 2013).

### Statistical Analysis

Statistical analyses were performed with GraphPad software using the Wilcoxon signed-rank test for paired nonparametric data. In some cases, Mann-Whitney test was used to compare unpaired samples.

## Results

### Classification of the Phenotype

Starting from the clinical data described above, we attempted to classify the phenotype of the two patients in typical or atypical AT variants, according to the method reported by (Fievet et al., 2019).

In Table 1, we report the criteria considered most useful for the classification: ataxia before the age of 8, loss of autonomous ambulation before the age of 15, oculomotor apraxia before the age of 15, ocular telangiectasia before the age of 15, low plasma IgA levels and clinical immunodeficiency. From the clinical data at our disposal, both patients maintained the ability to walk autonomously longer than the typical AT patients, who usually lose their autonomous ambulation by the end of the first decade of life (Rothblum-Oviatt et al., 2016). Moreover, both patients suffered from a milder than usual oculomotor apraxia. From the immunologic point of view, patient AT1-54000 did not suffer from clinical immunodeficiency although IgA deficiency and lymphopenia have been documented. In contrast, patient AT1-54001 presented clinical immunodeficiency, lymphopenia, and discordant IgA values. Finally, both brothers did not show ocular telangiectasias. We calculated the percentage of positive criteria to classify the phenotype as “typical” or “atypical” as described (Fievet et al., 2019), i.e., the phenotype is considered typical only if it exceeds 50% of the positive criteria, otherwise it is considered atypical. According to this classification, both brothers could be considered atypical, showing a percentage of positive criteria of 25 and 40%, respectively.

**TABLE 1:** Phenotype classification according to Fievet et al., 2019.

Patient	LCL	Ataxia <8 years	Loss of walking <15 years	Oculomotor apraxia <15 years	Ocular telangiectasia <15 years	IgA deficiency	Clinical immunodeficiency	% Criteria	Phenotype classification
Older brother	AT1-54000	NA	No	NA	No	Yes	No	25	Atypical
Younger brother	AT1-54001	NA	No	Yes	No	No	Yes	40	Atypical

### *In silico* Analyses

#### Mutation Prediction

To investigate the possible effects of the c.7157C > A mutation at the gene, transcript, and protein levels, we first performed *in silico* analyses by Mutation Taster that gave a “disease causing” return with a *p* value of 0.99. The variant was found neither in ExAC nor in 1000G. The most prominent features of the analysed alteration were 1) amino acid sequence changed, 2) protein features (might be) affected, 3) splice site changes.

The predictive analysis showed that the c.7157C > A alteration is located at the chromosome position chr11: 108199815C > A, which corresponds to the nucleotide position c.7157C using the transcript NM_000,051.4 as reference sequence, and lies in the coding region for the FAT domain (Bosotti et al., 2000). At the protein level, the mutation leads to the production of a full-length ATM protein carrying a single amino acid substitution at position 2386; in particular, the missense mutation causes the replacement of the alanine residue (Ala, A) with a glutamate residue (Glu, E). According to the Grantham Matrix (score range from 0.0 to 215 (Grantham, 1974)), this amino acid change prediction is associated with a score of 107, which is considered an intermediate-high value for the difference between the two amino acids. Moreover, the software reported a very high level of conservation for the 2386 site, being the values of phastCons and phyloP 1 and 6.188, respectively (phastCons varies between 0–1, while phyloP ranges from −14 to +6). In detail, the software reported the match “all identical” for the 2386 position in Pan troglodytes, Mus musculus, Takifugu rubripes, Drosophila melanogaster and *Xenopus tropicalis*, indicating a high degree of conservation of the Ala residue. Related to protein features, the prediction software indicated that the Ala-Glu substitution would affect the FAT domain impairing kinase domain and its activity. Overall, this predictive analysis indicates that the mutation determines an amino acid substitution in the FAT domain (p.Ala2386Glu) and this missense mutation may influence the functionality of the PI3K kinase domain causing the disease.

Next, we compared this mutation prediction outcome with those obtained by other types of software (i.e., PolyPhen-2, PROVEAN, SIFT, and mutation assessor) and all of them predicted that the alteration would be probably damaging, deleterious, or damaging.

As last point, Mutation Taster showed that the variant might influence the primary transcript by splicing modification. This possibility was investigated by using NNSplice as algorithm that predicted the gain of a new donor splice site at position 7154 in the transcript NM_000,051.4, with a confidence score of the newly created splice site greater than 0.3 (score 0.67, range 0–1). To evaluate the possible creation of a donor or acceptor splice site and exonic splicing enhancer (ESE) or silencer (ESS), we used specific predictors such as MaxEntScan, SpliceAI and Human Splicing Finder (HSF). In contrast with Mutation Taster prediction, these types of software reported a very low possibility that the gained donor splice site would be actually used *in vivo*. In particular, MaxEntScan, based on the Maximum Entropy model, reported a little decrease of the value for the mutated sequence (score -20.39 for the mutated sequence vs -25.91 for the WT). SpliceAI, based on deep learning approach (Jaganathan et al., 2019), found some other splice sites surrounding the mutation but with very low *p* value (∼0.03). Finally, HSF predicted several alterations of the auxiliary sequences and a significant alteration of the ratio of the ESE/ESS motifs.

In conclusion, the *in silico* predictions suggest that p. Ala2386Glu variant is pathogenic owing to two different hypotheses: it might affect protein features and activity or it might alter the splicing process, although the comparative analysis on the splicing alterations is not uniform.

#### Multiple Alignment

**FIGURE 1:**
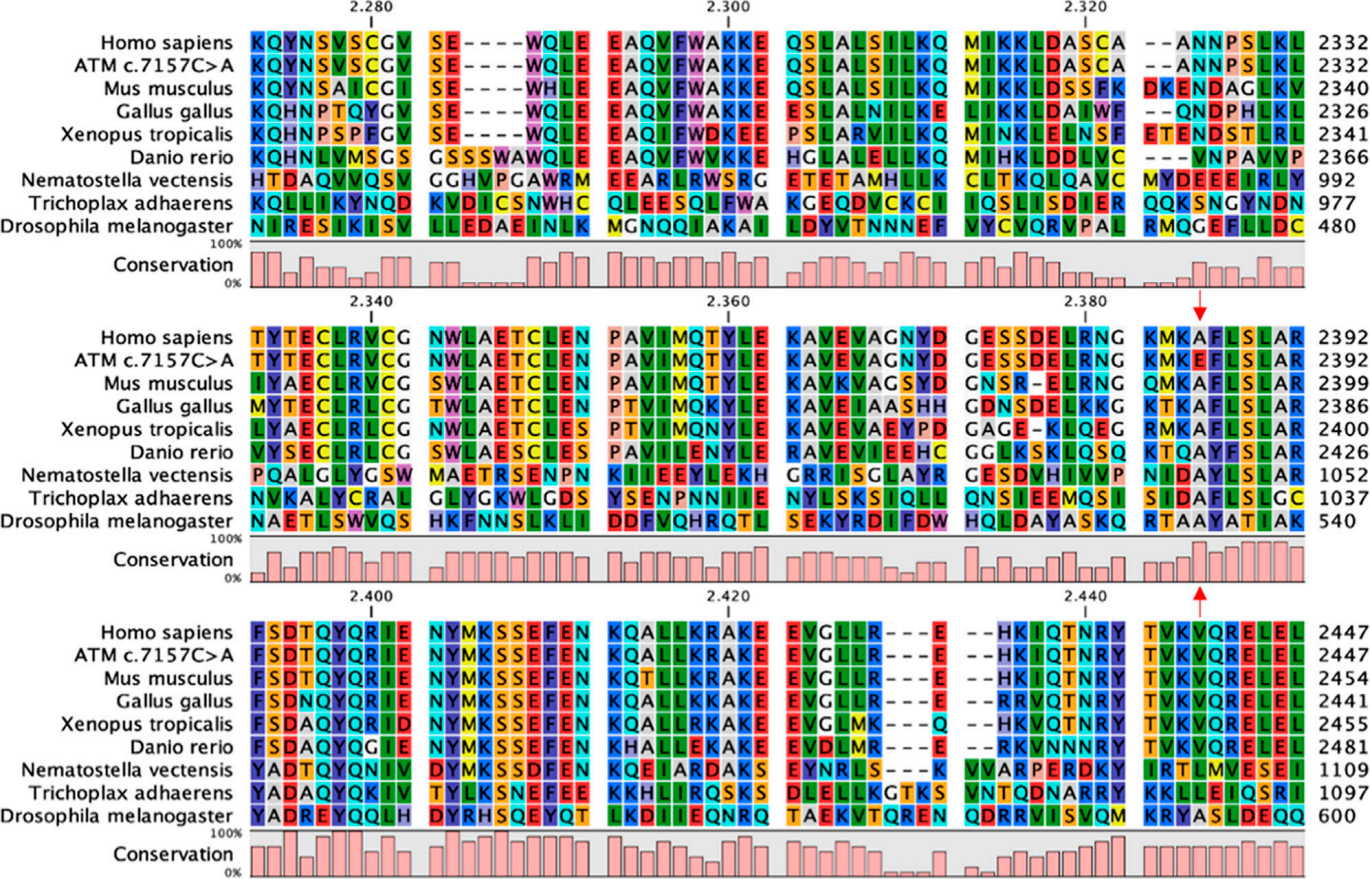
FAT domain conservation of ATM. Portion of the multiple sequence alignment of ATM orthologous sequences performed with CLC Sequence Viewer corresponding to the FAT domain. Red arrows indicate the Ala2386, completely conserved in the considered sequences, and the corresponding non-conservative mutation in the c.7157C > A variant.

To further investigate phylogenetic conservation of the mutated residue, multiple sequence alignment was performed with the CLC Sequence Viewer software. The FAT domain plays an important structural role by enveloping the kinase domain and affecting the activity of the whole protein (Baretic et al., 2017). Indeed, it is highly conserved along the evolutionary scale (Figure 1) and the position 2386, affected in our variant, is completely conserved in all the analysed sequences. In addition to our results, more complex alignments reported in the literature show that the first substitution of the Ala2386 with a valine residue appears in *Caenorhabditis elegans* and *Saccharomyces cerevisiae* (Baretic et al., 2017). This substitution is probably conservative, since both Ala and Val are aliphatic residues and share similar physicochemical properties and sizes. Ala conservation is lost when considering paralogous protein or more distant orthologous of human ATM and, in the corresponding position, non-conservative substitutions with aromatic or positively charged amino acids are found, such as tryptophan (in *Arabidopsis thaliana*) and histidine (in human mTOR) (Baretic et al., 2017).

#### Structural Prediction

To better predict the effect on protein structure of the p. Ala2386Glu variant, we analysed it by Missense3D and DynaMut software using, as template structure, the wild-type PDB ID 6K9L (Xiao et al., 2019), i.e., the only ATM structure in PDB with a resolution (4.27 Å) that permits to solve and recognize the residue side chains. In addition, we considered the PDB ID 5NP0 obtained by Baretic and collaborators (Baretic et al., 2017), since they reported an accurate analysis of ATM dynamics. The cryo-EM structure solved by Baretic shows that ATM protein is in a dynamic equilibrium between “closed” and “open” dimers. In the closed state, the PI3K regulatory domain blocks the peptide substrate-binding site, suggesting that this conformation may represent an inactive or basally active enzyme. The active site is held in the closed conformation by interaction of the kinase domain of one monomer with the TRD3 (tetratricopeptide repeats domain 3) of the FAT domain of the other monomer (Baretic et al., 2017).

**FIGURE 2:**
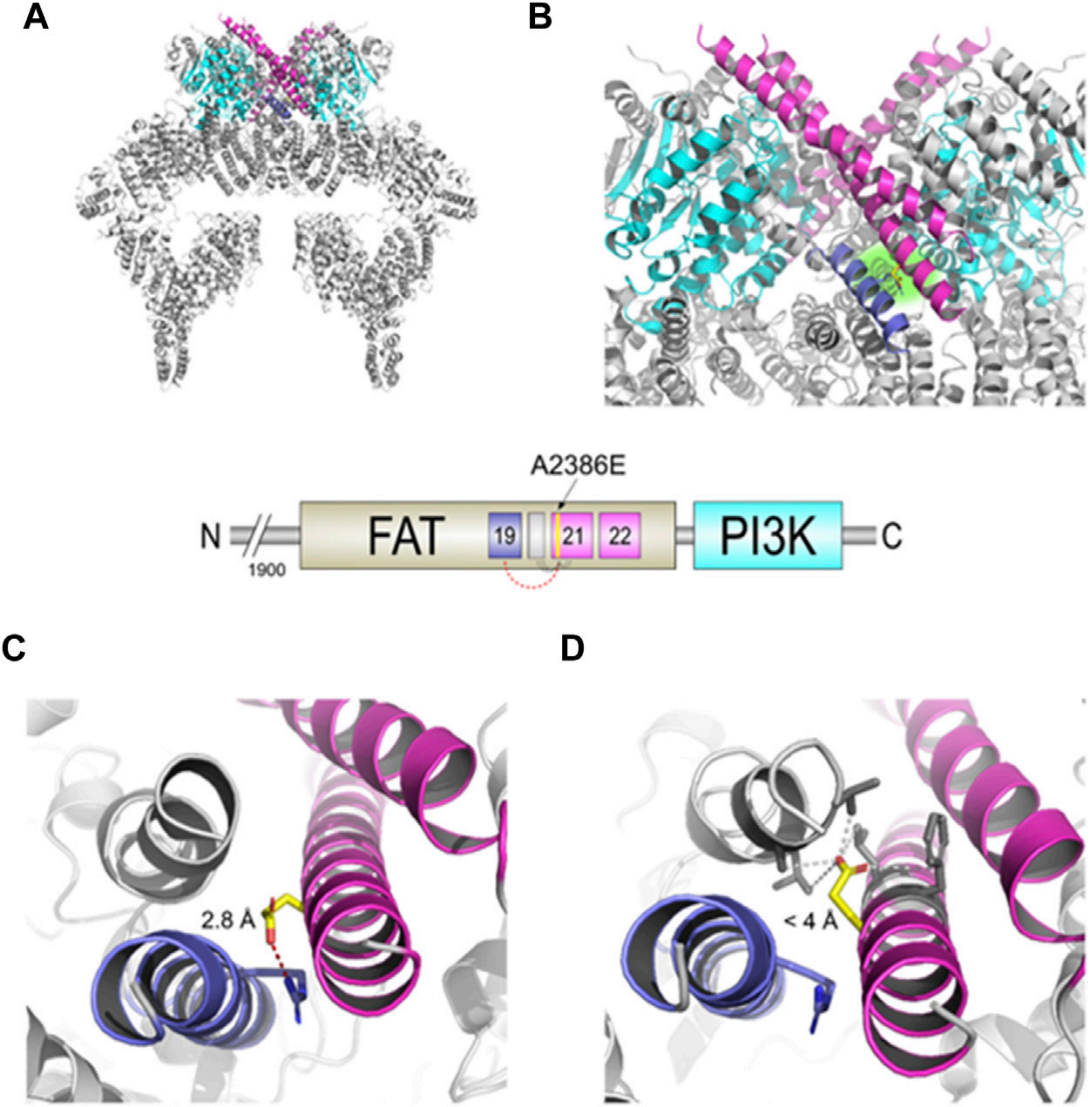
Structural changes prediction of the A2386E ATM mutated protein. The scheme in the centre shows the C-terminal FAT and PI3K domains of ATM with the colour legend of the *a* helices and domains used in figures. **(A)** Structural model of the mutated ATM obtained with Missense3D using PDB ID 6K9L as template. **(B)** Zoomed-in catalytic domains of the protein; Glu2386 is highlighted in yellow sticks and with a green-faded background. **(C)** Structural detail of A2386E predicted by DynaMut. Red-dashed line shows the distance of 2.8 Å between Glu2386 and Arg2339. **(D)** Structural detail of the mutant A2386E predicted by Missense3D. Grey-dashed lines show the distances <4 Å between Glu 2386 and the hydrophobic neighbour.

The c.7157C > A mutation lies in the α21 helix of the TRD3 of the FAT domain (Figures 2A,B) that, together with the similar α22, directly interacts with the PI3K domain of the other dimer subunit and actively participates in the transition between the “closed” and “open” conformation. The structural model obtained with DynaMut reveals a possible interaction between the carboxylic group of Glu2386 residue of the mutated protein and the guanidine group of Arg2339 residue on the α19 helix, with an inter-residue distance of only 2.8 Å (Figure 2C). The software predicted a stabilizing effect of the p. Ala2386Glu variant (ΔΔG: 2.085 kcal/mol) and decrease of molecule flexibility (ΔΔSVib ENCoM: 4.352 kcal.mol-1. K-1). This could result in a reduced activity of the whole protein due to stabilization of the closed conformation, preventing crucial rotation of the kinase-binding element (helices α21-22) in the close-to-open transition. In contrast, the structural model obtained with Missense3D showed a different orientation of the glutamate side chain, opposite to the DynaMut model, that is placed in a hydrophobic neighbourhood with the furthest residue at 4 Å (Figure 2D). The software also predicted “Structural damage detected” due to “buried hydrophilic introduced” and “buried charge introduced.”

Summarizing, our *in silico* analyses suggest that the p. Ala2386Glu ATM variant (from hereon, ATM-A2386E) may be pathogenic for two main but opposite reasons: 1) the amino acid variant might lead to the production of a partially active protein with reduced kinase activity due to a stabilization of the closed and less active dimer (according to DynaMut prediction, panel C) or 2) the variant might be responsible of a structural damage and a more unstable protein due to a buried hydrophilic/charge introduction (according to Missense3D prediction, Figure 2D).

### *In vitro* Identification and Quantification of Ataxia Telangiectasia-Mutated

**FIGURE 3:**
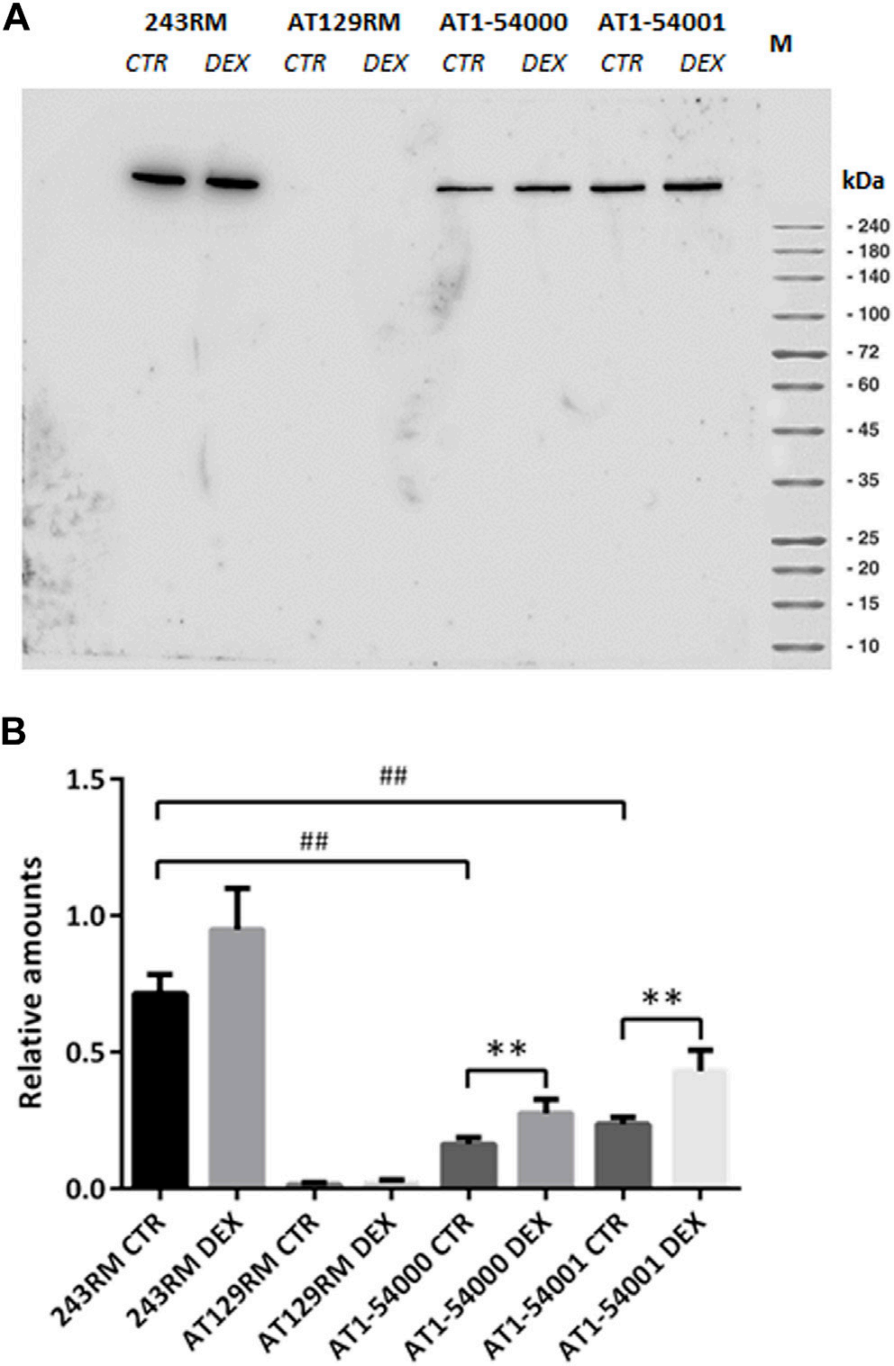
Protein quantification in LCL extracts. **(A)** Western blot analysis of ATM protein in WT (243RM), classical AT (AT129RM), AT1-54000 and AT1-54001 cells treated with 100 nM DEX for 24 h or not treated. **(B)** Quantification of the relative amounts of ATM protein in the total cell extracts of WT and AT cells shown in A. Blots shown are representative and the values are the means and SEM of four independent experiments. Statistics has been performed by comparing the WT CTR sample with the mutant CTR samples (Mann-Whitney test, ## two-tailed *p* values <0.005) or by comparing the same LCL without or with DEX treatment (Wilcoxon signed rand test; **two-tailed *p*-values<0.005).

As first *in vitro* investigation, we carried out Western blotting of protein extracts to assess the ATM protein in AT1-54000 and AT1-54001 LCLs. Similar analyses were performed on 243RM LCL carrying WT-ATM and AT129RM LCL carrying a homozygous truncating ATM mutation (Menotta et al., 2012), used as positive and negative controls, respectively. All the analyses were performed on cells untreated or treated with DEX. Figure 3A shows a representative blot of the ATM protein expressed in the investigated cells with and without DEX treatment. As expected, the full-length ATM protein was detected in the WT-ATM cells but not in the ATM-null cells. Consistent with the missense mutation, the ATM-A2386E protein was also present in both AT1-54000 and AT1-54001 cells. The quantification revealed that, in basal condition, ATM-A2386E protein expression is significantly lower than the WT-ATM protein (23 and 33% in the AT 1–54,000 and 1–54,001, respectively) (Figure 3B). This might be due to either a lower mRNA expression or a lower protein stability.

Upon DEX treatment, we observed a significant increase in ATM expression, both in AT1-54000 and AT1-54001 LCLs, reaching a fold change of about 1.7 and 1.8, respectively, compared to the untreated counterparts. Similar analysis was repeated after 48 h of stimulation confirming the induction, which was maximum at 24 h (Supplementary Figure S1). These results suggest that DEX is able to increase the expression of ATM-A2386E protein, rising the protein level to about 39 and 60% compared to the basal level of the WT-ATM control.

### Studies at mRNA Level

**FIGURE 4:**
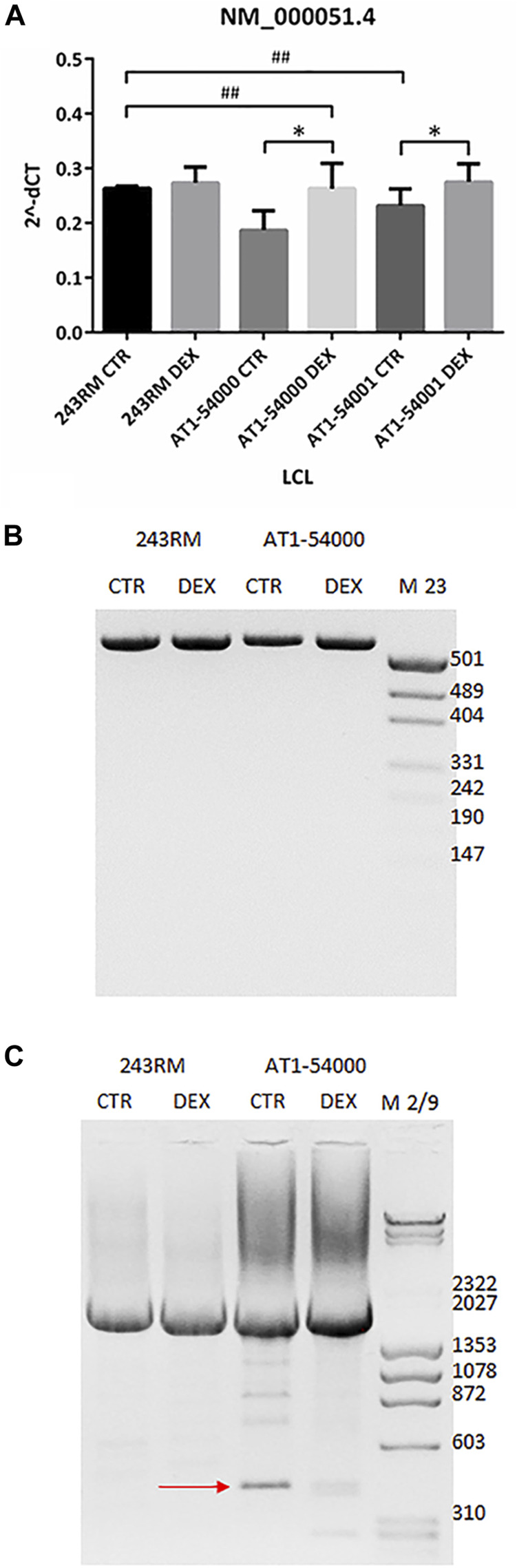
mRNA quantification in LCL extracts. **(A)** The native transcript NM_000,051.4 has been quantified by Real-Time PCR on the two mutated LCLs and on a WT cell line used as control. Values are means and SEM of four independent experiments; statistics has been performed by comparing the WT CTR sample with the mutant CTR samples (Mann-Whitney test, ## two-tailed *p* values <0.005) or the same sample without or with DEX treatment (Wilcoxon signed rand test; *two-tailed *p*-values<0.05). **(B)** Alternatively-spliced transcript has been investigated by end-point PCR on the same cDNA used in panel B. Forward primer anneals at the start of exon 48 while reverse primer at the end of exon 50. Native band = 540 bp, alternatively spliced band = 340 bp, according to mutation taster prediction. **(C)** End-point PCR has been performed as in panel B but using as reverse a primer annealing to the end of exon 63 (last exon). Native band = 2195 bp, alternatively spliced band = 423 bp. The newly identified alternatively spliced band sequencing is reported in Supplementary Figure S2.

To investigate whether the reduced ATM protein expression observed in the mutant LCLs is due to reduced gene expression and/or to an alternative splicing event, as predicted by Mutation Taster, we performed some experiments at the mRNA level. First, we quantified the level of the native ATM transcript (NM_000,051.4) by quantitative Real-Time PCR. As shown in Figure 4A, we found significantly lower levels of native transcript in the patient’s LCLs compared to WT cells (*p* value 0.0286 and 0.0333 for AT1-54000 and AT1-54001, respectively, calculated by Mann-Whitney test; asterisks not shown in the figure), supporting the hypothesis of an alternative splicing event. Indeed, based on the predicted new donor splice site, the splicing event would cause a deletion of the last 154 nucleotides of exon 49 bringing the translation of the protein out of frame at position 2387 in the protein sequence. Moreover, the putative alternative splicing might also affect the final concentration of the native transcript. To test this possibility, we attempted to identify alternative-splicing event(s) by end-point PCR (Figure 4B). Amplicons were obtained by amplifying the cDNA from the beginning of exon 48 to the end of exon 50. Native transcript is expected to yield a band of 540 bps while the predicted alternative spliced transcript would yield a band of 340 bps. By this method, only the main band corresponding to the native transcript was detected in the tested LCLs. Thus, we tried another strategy by amplifying the last exons of ATM. In particular, the forward primer anneals at the beginning of exon 48 and the reverse primer at the end of the last exon (exon 63). As shown in Figure 4C, with these primers we identified a faster migrating band in the AT1-54000 LCLs that is not present in the WT cells. In addition, the presence of this band was reduced by DEX treatment together with an increase in the levels of the native transcript. Next, by cloning and sequencing this new band, we showed that it derives from a non-canonical splicing in exon 49 generating a deletion of the last part of the exon. The exon/intron boundary is about 46 nucleotides upstream the site predicted by NNSPlice and join together the first 18 bps of exon 49 with the last 106 bps of exon 62 (Supplementary Figure S2). In summary, this new transcript could, at least in part, account for the decrease of the native transcript observed in the mutant LCLs and ultimately for the reduced protein amount.

### Studies at Protein Level

To investigate whether the low ATM-A2386E protein amount might be due to reduced protein stability, as predicted by Missense3D (Figure 2D), we compared the stability of WT-ATM and ATM-A2386E proteins by incubating WT-ATM 243RM and AT1-54001 LCLs at increasing temperatures (from 37°C to 44°C) before performing a Western blot. This analysis was performed in basal conditions and after DEX treatment. As shown in Supplementary Figure S3, the amount of ATM-A2386E protein was strongly reduced already at 42°C while the WT protein was still present at 44°C, suggesting that ATM-A2386E has reduced protein stability. Of relevance, consistent with induction of ATM protein driven by the heat shock response, WT-ATM protein was induced at 40° and 42°C, while ATM-A2386E was not. Finally, DEX treatment did not affect protein stability (Supplementary Figure S4). Overall, these results agree with the Missense3D prediction of loss of stability due to the introduction of a Glu residue (polar, negatively-charged, and with a greater steric hindrance) in a buried hydrophobic pocket of the protein.

### Activity and Functional Assays

As final point, we investigated the activity of ATM-A2386E protein by performing two functional assays based on ATM kinase activity. The analyses were performed on LCLs before and after DEX treatment.

#### CHK2 Activation (ATM Activity Assay)

**FIGURE 5:**
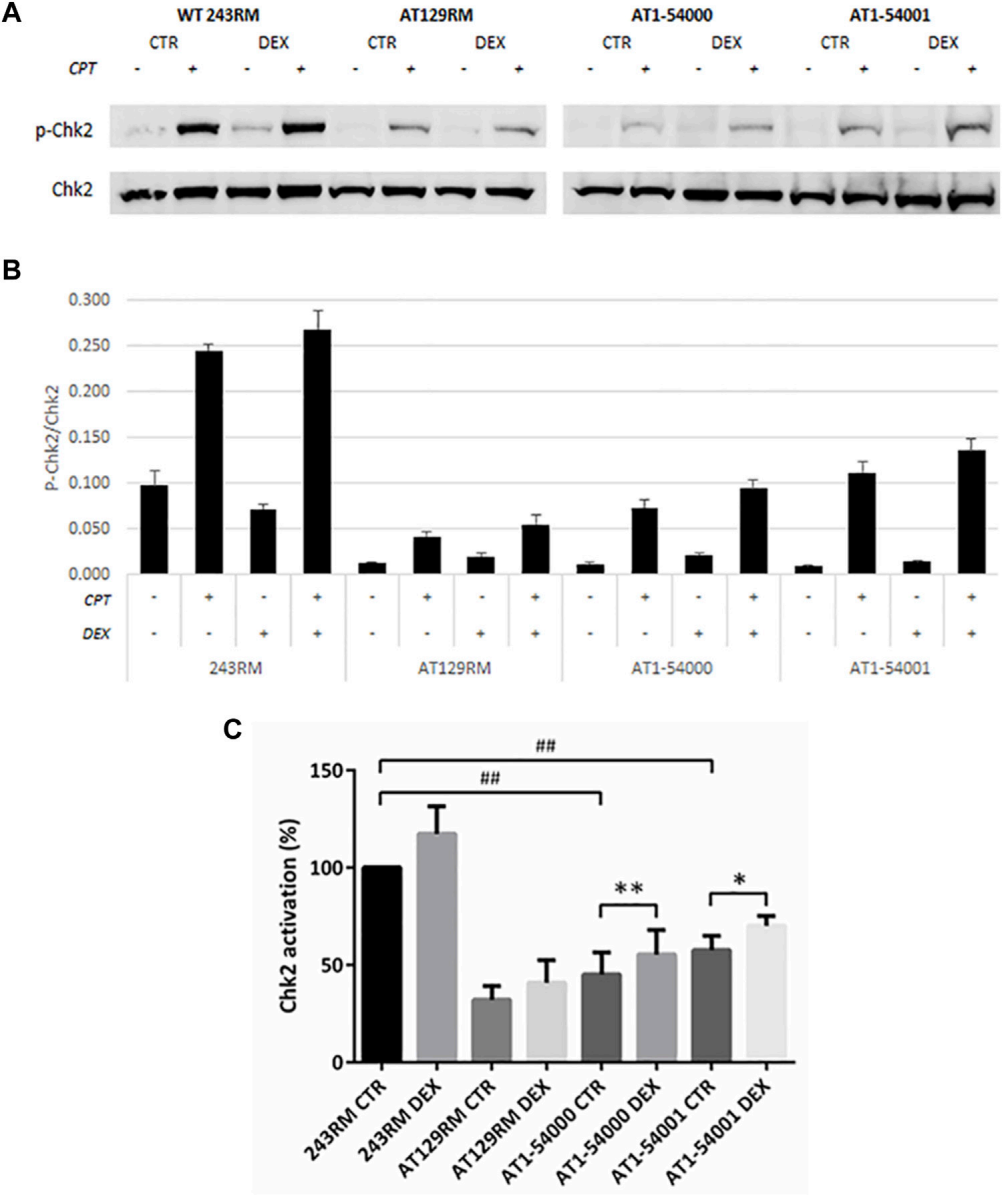
Chk2 activation in LCL extracts. The activation status of Chk2 has been investigated in WT and AT cells, treated or not with DEX, and with or without CPT treatment. **(A)** Western blot analysis of *p*-CHK2 (Thr68) and total Chk2 protein in WT (243RM), classical AT (AT129RM), AT1-54000 and AT1-54001 protein extracts. **(B)** Quantification of the relative amounts of *p*-Chk2/Chk2 in the cell extracts of WT and AT cells shown in A. Blots shown are representative and values are the means and SEM of four independent experiments. **(C)** Quantification of Chk2 activation in each cell line, treated or not with DEX. Quantification has been obtained by subtracting the *p*-Chk2/Chk2 level found in the samples untreated with CPT from the respective sample treated with CPT. Values have been then expressed as percentage with respect to the WT control assumed as 100%. Significance has been calculated by using the non-parametric Mann-Whitney test for unpaired samples (## two-tailed *p* values <0.005) or by using the Wilcoxon signed rand test for paired samples treated or not with DEX (two-tailed *p*-values *<0.05, **<0.005).

First, we assessed the activity of ATM by analysing the phosphorylation of a well-known ATM target, the protein kinase CHK2, in the absence and presence of CPT for ATM activation. As expected, a conspicuous phosphorylation of CHK2 was detected in WT-ATM LCLs (Figure 5); interestingly, a lower but still significant CHK2 phosphorylation was present in AT1-54000 and AT1-54001 LCLs, suggesting that ATM-A2386E protein maintains, at least in part, its kinase activity. Surprisingly, a slight CHK2 phosphorylation was also observed in the ATM-null AT129RM cells. This activity has been attributed to ATR protein function (Fievet et al., 2019) and/or to the presence of a mini-ATM that compensates for the absence of the full-length protein (Menotta et al., 2012). Next, we quantified the activation of CHK2 in each sample by normalizing the phosphorylation levels of CHK2 after CPT with those present at the basal level (i.e., the same LCL without CPT). In the histogram reported in Figure 5C, quantification was expressed as the percentage of activation compared to basal WT sample used as control. In the absence of DEX, we calculated that the percentage of activation in AT1-54000 and AT1-54001 LCLs is significantly lower than the WT control (about 40 and 60%, respectively). Furthermore, we observed that DEX was able to significantly increase the activation rate in both AT1 LCLs, consistent with induction of ATM-A2386E protein expression. In summary, together with the lower amount of ATM-A2386E protein compared with the WT reported in Figure 3, these results suggest a fully active protein (less abundant) rather than a partially active protein (normally expressed).

#### p53 Mitotic Centrosome Localization

**FIGURE 6:**
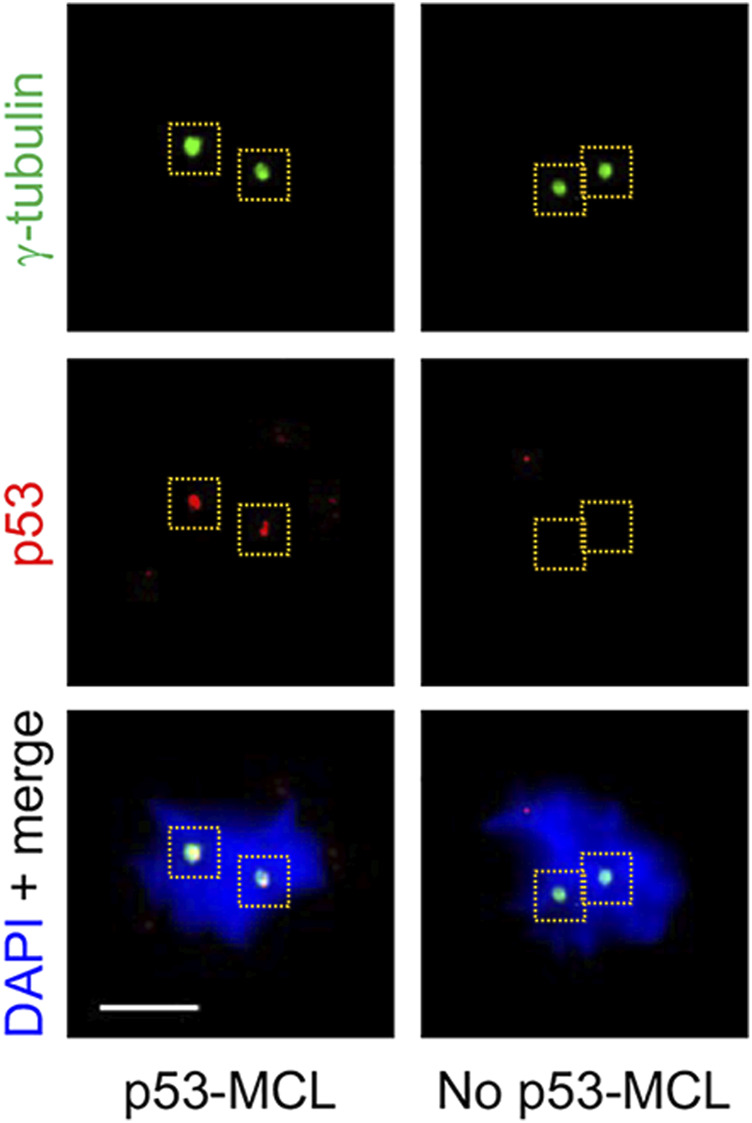
p53-MCL in AT1-54000 and AT1-54001 cell lines. Representative immunofluorescence images of p53-MCL in LCLs. In particular, the AT1-54000 non-treated cells are shown. Cells were stained for p53 (red) and y-tubulin (green) for centrosome identification (yellow dashed squares). DNA (blue) was stained with DAPI. p53-MCL was evaluated by counting mitotic cells showing (p53-MCL) or not (no p53-MCL) p53-centrosome colocalization. Scale bar = 5 µm, ×100 objective.

To further investigate the activity of the ATM-A2386E protein, we carried out a different functional assay that still requires ATM kinase activity (i.e., p53-Ser15 phosphorylation) but is independent of the well-characterized DNA damage response employed above (i.e., CPT-induced CHK2 phosphorylation). This assay is based on the ATM-dependent localization of the p53 tumour suppressor at the centrosomes during mitosis (p53-MCL) (Prodosmo et al., 2013; Contadini et al., 2019). The assay is able to discriminate healthy individuals (i.e., WT-ATM alleles; p53-MCL >75%) from typical AT patients (i.e., biallelic ATM mutations; p53-MCL <30%) and from AT healthy carriers (i.e., monoallelic ATM mutations; p53-MCL >40% < 65%) (Prodosmo et al., 2013). Of relevance, inhibition of p53-MCL has been also observed in breast cancer patients carrying monoallelic, missense ATM variants (Prodosmo et al., 2013; Prodosmo et al., 2016). At variance with other ATM diagnostic tests, the p53-MCL assay does not measure a continuous quantitative variation (e.g., radiosensitivity, ATM protein levels, and phosphorylation of ATM targets) but a “binary” outcome. Indeed, at the single cell level, p53 does or does not localize at the centrosomes while, at the cell-population level, the number of cells showing one or the other phenotype allows to unambiguously diagnose not only AT homozygotes, but also AT heterozygotes (Prodosmo et al., 2013). When this test was carried out on AT1-54000 and AT1-54001 LCLs (Figure 6 and data not shown), we observed a percentage of p53-MCL (62 and 48%, respectively) that was compatible with a heterozygous profile (Supplementary Figure S5, grey columns) indicating that ATM-A2386E partially maintains the capacity to promote p53-MCL. However, DEX treatment did not significantly improve the percentage of p53-MCL and the cells maintained the heterozygous profile (Supplementary Figure S5). Together with the data obtained above by the classical ATM kinase activity assay, these results suggest that ATM-A2386E protein preserves, at least in part, its kinase activity.

### Variant Interpretation

Finally, we applied the data described above to the guidelines of the American College of Medical Genetics and Genomics and the Association for Molecular Pathology (ACMG/AMP) for the interpretation of sequence variants (Richards et al., 2015). As reported in Supplementary Table S2, our variant satisfies several evidences of pathogenicity, such as one strong, two moderate, and four supporting. Thus, based on the rules for combining criteria, the ATM c.7157C > A variant can be classified as pathogenic.

## Discussion

Based on the Human Gene Mutation database (HGMD) that collects germline mutations underlying or associated with human inherited diseases (Stenson et al., 2014; Stenson et al., 2020), 1,924 ATM variants have been described of which 756 are associated with AT. However, more than 10,000 ATM variants have been reported in the genetic variation database ClinVar, of which 1,485 are clearly pathogenic, 661 likely pathogenic, 304 benign, and 2,496 likely benign, while 5,209 are classified as VUS and 500 with conflicting interpretations (https://www.ncbi.nlm.nih.gov/clinvar, last update September 29th, 2021). These data highlight the existence of a still large number of variants with pending disease association for the inclusion in the HGMD collection.

Thus far, most of the variants classified as pathogenic are nonsense or frameshift mutations, whereas most of the VUS are missense variants. This raises the need to clarify the possible pathogenicity of these missense variants and, at the same time, the availability of functional assays that can predict the associated phenotype. The present work is aimed to classify the ATM c.7157C > A variant as pathogenic or not and to assess the potential benefit of DEX treatment.

The ATM c.7157C > A variant was submitted for the first time in ClinVar in 2016, classified as associated with cancer-predisposition syndrome, and in 2019 associated with AT (RCV000627922.1). However, it is still classified as VUS because the available evidence is currently insufficient to determine its pathogenic role. Our *in silico* analyses showed that the c.7157C > A variant is not present in ExAC, 1000G, and COSMIC, but it is identified in dbSNP database as a VUS and with the following allele frequency: A = 0.000004 (1/251,314, GnomAD_exome), A = 0.00003 (1/31,376, GnomAD_genomes) and A = 0.0 (0/10,680, ALFA Project). LOVD is the only database in which the alteration is classified as pathogenic (#0000474464) (Fievet et al., 2019). When we began this work, the group of Fievet published a paper on the functional classification of atypical AT variants. The study focused on 49 variants: 17 classified as pathogenic, 8 classified as mildly pathogenic, and 24 as VUS. The variant c.7157C > A identified in a patient with atypical phenotype was included among the VUS (Fievet et al., 2019).

Our work started with the clinical phenotype observed in two brothers of Lebanese origin, carriers of the c.7157C > A variant in homozygosity. Both patients developed severe walking impairment only after the age of 15; they did not present ocular telangiectasia and one of them showed oculomotor apraxia (for the older patient no data are available). Regarding the immunological status, one patient showed IgA deficiency while the other clinical immunodeficiencies. Following Fievet’s criteria, both patients were classified as having an atypical phenotype, being the percentage of positive criteria under 50%.

Next, we carried out *in silico* studies and predicted that the c.7157C > A variant causes an amino acid change, A2386E, within the highly conserved FAT domain and might produce the gain of a new donor splice site few nucleotides upstream the point mutation.

Further *in silico* alignments performed with homologous sequences of some landmarks of the eukaryotic crown-group Opisthokonta underscored a high conservation of the amino acid at position 2368. Indeed, going back along the evolutionary scale, the first substitutions are found in *Caenorhabditis elegans* and *Saccharomyces cerevisiae* where, nonetheless, the Ala residue is substituted with Valine, an amino acid with similar properties.

To understand how the c.7157C > A missense mutation in the FAT domain may affect protein structure and activity, we performed structural analyses by comparing the prediction of the mutant protein with the wild-type one. WT-ATM protein is mainly found in two different conformations: “closed dimer” (with lower enzymatic activity) and “open dimer” (with higher enzymatic activity) (Baretic et al., 2017). In the closed conformation, a PRD regulatory domain (PI3K Regulatory Domain) acts as a pseudo-substrate and inhibits the access of the substrate to the active site. Instead, in the open conformation the active site is free to bind the substrate without hinder. The closed dimer has the shape of a butterfly, where the upper part (head) consists of the so-called FATKIN domain (aa 1903–3056), which results from the joining of FAT and KIN (kinase) domains. The FATKIN domain mediates ATM dimerization and is therefore relevant for the protein activation. It consists of five subdomains: TRD 1-3, HRD, and KIN. The TRD3 (aa2195-2476, α13-22) in which our A2386E mutation lies is predicted to form important *trans*-molecular interactions with the kinase domain of the other dimer subunit (see Figure 2B). Key elements have been identified for the transition between the closed and open dimer which include FATC, LBE, Activation loop and PRD domains; all these elements give rise to a regulation system called FLAP. In particular, the helices α21-22 of TRD3 (residues 2378–2476, in which the 2386 residue lies) interact with all the elements of the FLAP (they are also referred to as FLAP-Binding Element or FLAP-BE). The FLAP-BE of one subunit pushes on the α helices kα9c and kα9d (residues 2966–2979) in the PRD of the other subunit, and therefore is very important for the stabilization of the closed conformation. Indeed, the interaction of the helices α21-22 with the α16-18 of the same domain determines a rotation of about 24°, which leads to the transition from the closed conformation to the open one. The substitution of the Ala2386 residue with a Glu may likely interfere with the complex aforementioned mechanism. DynaMut analysis allowed us to identify a possible interaction of Glu2386 with Arg2339. The predicted Glu2386-Arg2339 electrostatic interaction could therefore stabilize the closed conformation, impeding the rotation of the pseudosubstrate and the resultant active site opening. In support of this hypothesis, Baretic found three PI3K-specific polar interactions between the FAT and KIN domains of the wild-type protein: Glu1959-Arg2849; Arg2486-Glu2950; Gln2522-Gln2730 (Baretic et al., 2017). These residues, like Ala2386, are highly conserved and mutations in these sites are considered pathogenic. For example, the Arg2849Pro mutation has been associated with AT (Sandoval et al., 1999) while the Arg2486Pro mutation with promyelocytic T-cell leukaemia (Stoppa-Lyonnet et al., 1998).

An alternative hypothesis has been suggested by the analysis with Missense3D. This software predicted a loss of protein stability due to the introduction of a hydrophilic amino acid (Glu) in a buried hydrophobic pocket affecting the whole protein stability. This is of particular interest because the replacement of a small hydrophobic residue with a larger hydrophilic negative one has been defined as a major cause of instability by Ittisoponpisan and collaborators within the missense variant they considered (Ittisoponpisan et al., 2019). In addition, by measuring the statistical burden of variations, Iqbal and others reported that kinases show a particular enrichment of pathogenic variants in α helical residues; interestingly, these residues are relatively buried with a probability of 74% in the class of kinase proteins (Iqbal et al., 2020). In any case, both hypotheses would lead to the production of a pathogenic variant owing to a minor protein abundance or to a minor protein activity.

After these *in silico* studies, we carried out *in vitro* studies to assess protein abundance and activity on the LCLs derived from the two patients. We found that the mutant full-length ATM protein is present in the LCLs from both brothers; however, its expression was significantly lower than the ATM in the WT LCLs (*p* value 0.0040) used as control. This result agrees with the observations made by Fievet et al. in the AT27 cells (Fievet et al., 2019), where they reported a level of mutant protein of about 30% of the WT protein. When we attempted to experimentally evaluate whether this low protein abundance might be due to an alternative splicing event or to a “buried mutation,” as predicted by different types of software, we found evidence supporting both models. Consistently with the alternative splicing model, we observed that levels of the native transcript are indeed significantly lower in mutated than in WT cells (Figure 4), suggesting that some pre-mRNA molecules might be alternative spliced and ultimately lead to a decrease of the native one. Indeed, we found a band corresponding to a non-canonical splicing event that joined the first 18 bases of exon 49 with the latter 106 nucleotides of exon 62, leading to a complete skipping the kinase domain. Moreover, *in vitro* translation showed that this newly identified transcript would generate a premature stop codon at residue 2370. Thus, the occurrence of this alternative splicing might partly account for the lower native mRNA and protein levels. This hypothesis is supported by the observation made by Eng et al., which demonstrated that the ATM gene possess weak splice sites that render the gene very susceptible to splice mutation and to physiological alternative splicing (Eng et al., 2004). By using MaxEntScan (based on the principle of maximum entropy) they revealed that three missense mutations in the ATM gene were indeed splicing mutation disrupting normal splice sites (Eng et al., 2004). In addition, it is noteworthy that although both ATM protein and mRNA levels were increased by DEX treatment, the results reported in Figures 3, 4 suggest that the effect of DEX is mainly played at the mRNA level by increasing the amount of native transcript and reducing the alternatively-spliced transcript.

Next, we investigated the hypothesis that the lower protein expression is due to reduced protein stability. In agreement with the “buried mutation” model, we observed that the mutant protein is less stable than the WT. Interestingly, during the *in vitro* stability experiment, we observed that WT-ATM protein is induced by heat shock stress, in agreement with previously reported data showing that ATM is also activated by heat shock independently of DNA damage (Hunt et al., 2007). However, in contrast to the changes we observed at the mRNA levels, protein stability was not appreciably modified by DEX treatment.

Finally, we investigated the *in vitro* activity of ATM-A2386E protein via two different functional assays. Both suggested that the mutant protein maintains, at least in part, the kinase activity, suggesting that the reduced activation observed in our *in vitro* studies might be due to a lower protein content rather than a lower protein functionality. This agrees with the observation of an activation capacity of ATM of approximately 68% in mutant cells compared to the WT (Fievet et al., 2019). Since CHK2 activation could be partly due to overlapping ATR activity, we performed a further immunofluorescence assay, p53-MCL (Prodosmo et al., 2013), which confirmed the functionality of the mutant ATM, at the same level of a monoallelic mutant ATM. Regarding the effect of DEX, we found a higher CHK2 activation in cells treated with the drug, consistent with the higher protein level, while we did not find an increased functionality in the p53-MCL test. Taken together, our results are more consistent with the hypothesis that the variant c.7157C > A causes a loss of stability, rather than a loss of functionality; this is confirmed by the presence, in the mutant cells, of a lower protein expression (23–33% compared to WT) but with an almost normal activity (40–60% compared to WT).

## Conclusions

In conclusion, our results suggest that the ATM c.7157C > A variant, far from being a “silent” missense variant, is pathogenic, also according to the ACMG/AMP criteria for variant classification. Indeed, the variant can affect the protein expression and activity and causes an atypical phenotype in the patients. We hypothesized that the lower protein expression and activity is due to loss of protein stability and, in part, to changes in the splicing process. Last but not least, we demonstrated that DEX treatment can exert a positive effect also on a missense variant responsible for atypical AT by increasing protein expression and activity. This is of particular importance in the light of the recently approved clinical trial for the treatment of AT by intra-erythrocyte DEX (ClinicalTrials.gov Identifier: NCT02770807).

## Data Availability

The datasets for this article are not publicly available due to concerns regarding participant/patient anonymity. Requests to access the datasets should be directed to the corresponding author.
