# Supplementary material for: Functional Classification of the ATM Variant c.7157C>A and In Vitro Effects of Dexamethasone
Source: Front Genet. 2021 Oct 25;12:759467. doi: 10.3389/fgene.2021.759467 (PMC8573154; doi:10.3389/fgene.2021.759467)
Supplement: Supplementary file 1 [file DataSheet1.docx]

# SUPPLEMENTARY MATERIAL

## Supplementary Table S1

**Primer pair 1**

|  | **Sequence (5'->3')** | **Template strand** | **Length** | **Start** | **Stop** | **Tm** | **GC%** | **Self-complementarity** | **Self-3' complementarity** |
| --- | --- | --- | --- | --- | --- | --- | --- | --- | --- |
| **Forward primer** | AACAATCCCAGCCTAAAACTTAC | Plus | 23 | 6976 | 6998 | 57.02 | 39.13 | 3.00 | 1.00 |
| **Reverse primer** | CTTCATCATGCCATTGACTTCAG | Minus | 23 | 7515 | 7493 | 58.08 | 43.48 | 5.00 | 3.00 |
| **Product length** | 540 | | | | | | | | |

**Primer pair 2**

|  | **Sequence (5'->3')** | **Template strand** | **Length** | **Start** | **Stop** | **Tm** | **GC%** | **Self-complementarity** | **Self-3' complementarity** |
| --- | --- | --- | --- | --- | --- | --- | --- | --- | --- |
| **Forward primer** | AACAATCCCAGCCTAAAACTTAC | Plus | 23 | 6976 | 6998 | 57.02 | 39.13 | 3.00 | 1.00 |
| **Reverse primer** | TCACACCCAAGCTTTCCATC | Minus | 20 | 9171 | 9152 | 58.09 | 50.00 | 6.00 | 0.00 |
| **Product length** | 2196 | | | | | | | | |

**Supplementary Table S1. List of primers.** The table displays the two pairs of primers used in the end-point PCR showed in Figure 4B and C. In both strategies, the forward primer has been designed at the beginning of exon 48 at position 6976; while the reverse primers were at the end of exon 49 and at the end of exon 63, respectively. The NM000051.4 transcript has been used as reference sequence.

## Supplementary Table S2

| **Evidence of pathogenicity** | | **Categories** | **Criteria satisfied** | **Notes** |
| --- | --- | --- | --- | --- |
| VERY STRONG | PVS1 | Null variant (nonsense, frameshift, canonical ±1 or 2 splice sites, initiation codon, single or multiexon deletion) in a gene where LOF is a known mechanism of disease | No |  |
| STRONG | PS1 | Same amino acid change as a previously established pathogenic variant regardless of nucleotide change | No |  |
|  | PS2 | De novo (both maternity and paternity confirmed) in a patient with the disease and no family history | No |  |
|  | PS3 | Well-established in vitro or in vivo functional studies supportive of a damaging effect on the gene or gene product | Yes |  |
|  | PS4 | The prevalence of the variant in affected individuals is significantly increased compared with the prevalence in controls | No |  |
| MODERATE | PM1 | Located in a mutational hot spot and/or critical and well-established functional domain (e.g., active site of an enzyme) without benign variation | Yes | *According to Varsome** |
|  | PM2 | Absent from controls (or at extremely low frequency if recessive) (Table 6) in Exome Sequencing Project, 1000 Genomes Project, or Exome Aggregation Consortium | Yes | *According to Varsome** |
|  | PM3 | For recessive disorders, detected in trans with a pathogenic variant | No |  |
|  | PM4 | Protein length changes as a result of in-frame deletions/insertions in a nonrepeat region or stop-loss variants | No |  |
|  | PM5 | Novel missense change at an amino acid residue where a different missense change determined to be pathogenic has been seen before | No |  |
|  | PM6 | Assumed de novo, but without confirmation of paternity and maternity | No |  |
| SUPPORTING | PP1 | Cosegregation with disease in multiple affected family members in a gene definitively known to cause the disease | Yes | *Affected brother* |
|  | PP2 | Missense variant in a gene that has a low rate of benign missense variation and in which missense variants are a common mechanism of disease | No |  |
|  | PP3 | Multiple lines of computational evidence support a deleterious effect on the gene or gene product (conservation, evolutionary, splicing impact, etc.) | Yes | *According to Varsome** |
|  | PP4 | Patient’s phenotype or family history is highly specific for a disease with a single genetic etiology | Yes |  |
|  | PP5 | Reputable source recently reports variant as pathogenic, but the evidence is not available to the laboratory to perform an independent evaluation | Yes |  |

**Supplementary Table S2. Criteria for pathogenicity.** The table shows the criteria used for variant classification according to ACMG Standards and Guidelines (41).

**VarSome: the human genomic variant search engine, Christos Kopanos, Vasilis Tsiolkas, Alexandros Kouris, Charles E Chapple, Monica Albarca Aguilera, Richard Meyer, Andreas Massouras, Bioinformatics, Volume 35, Issue 11, 1 June 2019, Pages 1978–1980, https://doi.org/10.1093/bioinformatics/bty897*

## Supplementary Figure S1


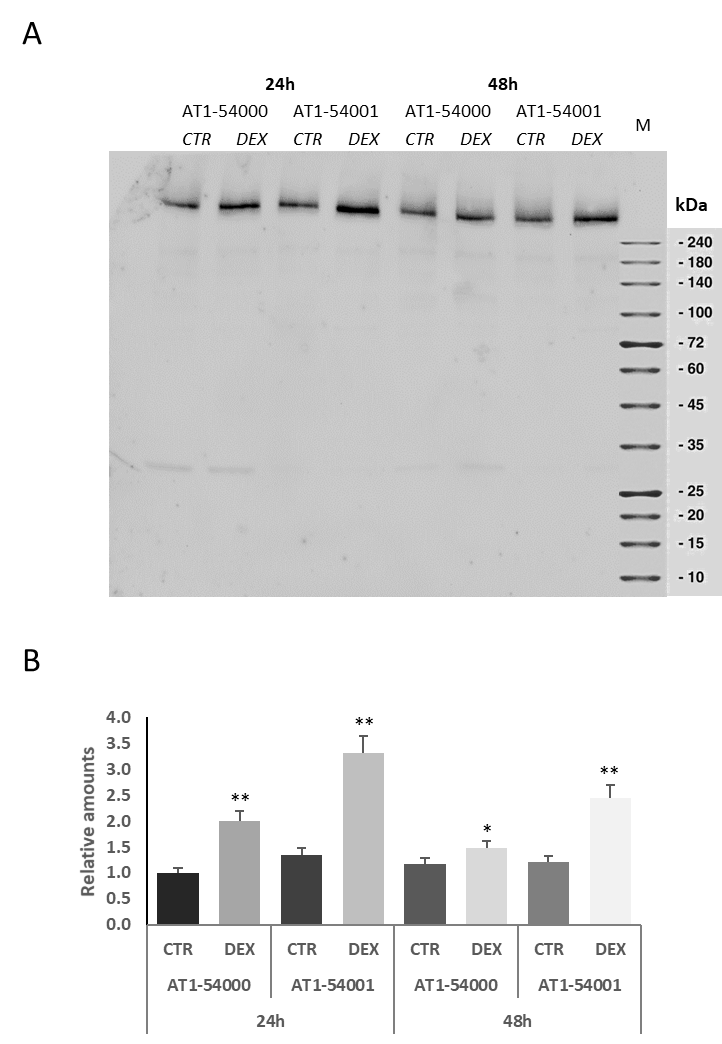


**Figure S1. Protein quantification in LCL extracts after 24/48h DEX.** (**A**) Western blot analysis of ATM protein in AT1-54000 and AT1-54001 cells treated with 100 nM DEX for 24/48 hours or not treated. (**B**) Quantification of the relative amounts of ATM protein in the total cell extracts of WT and AT cells shown in Blots shown are representative and values are the means and SEM of four independent experiments (Wilcoxon signed rand test; *two-tailed p-values<0.05).

## Supplementary Figure S2

*
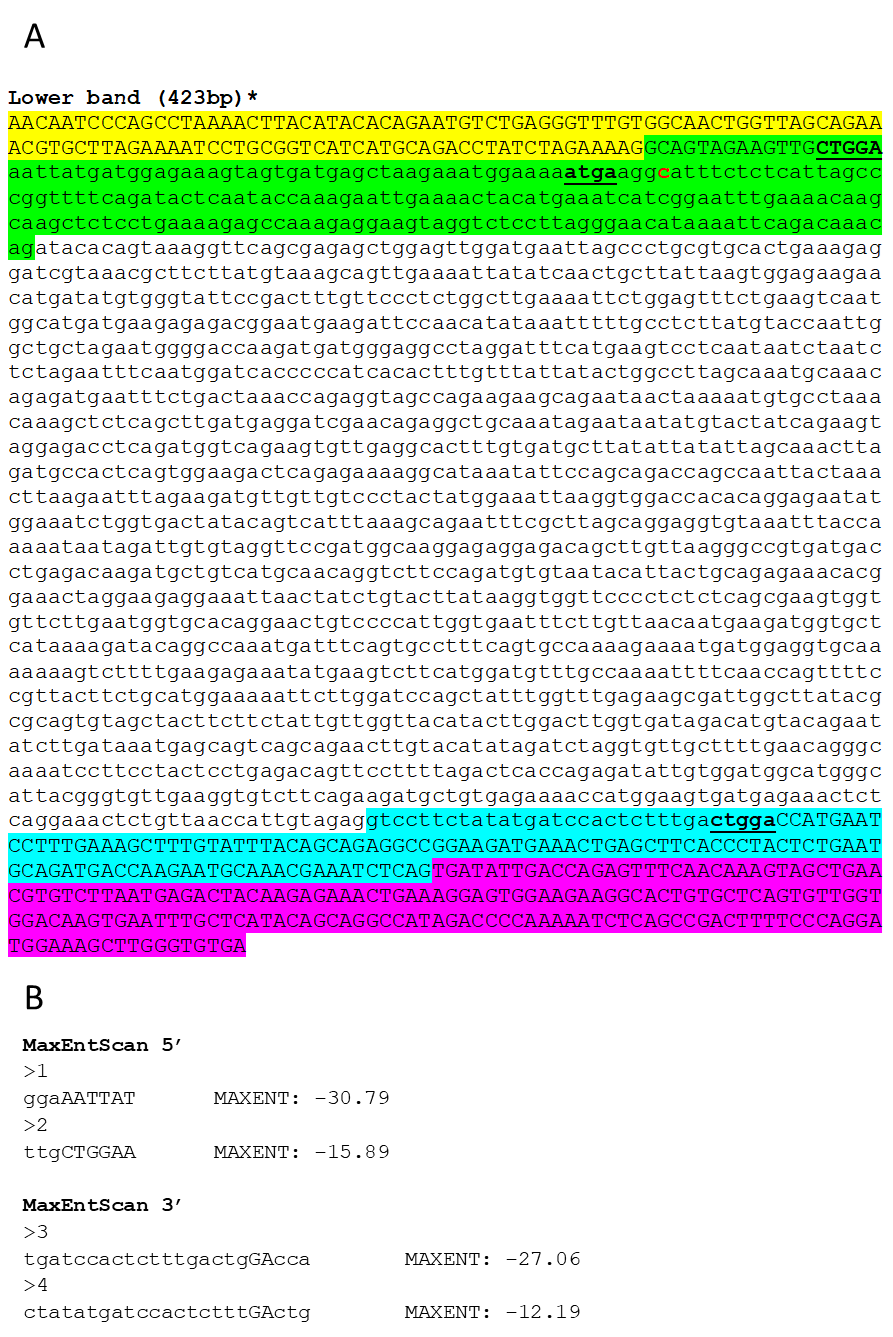
*

**Figure S2.** (**A**) **Sequencing of the lower band newly identified in Figure 4C.** The CDS of NM000051.4 has been used as reference sequence. *Yellow, exon 48; green, ex. 49; cyan, exon 62; magenta, exon 63. Uppercase, exonic sequences; lowercase, intronic sequences. Underlined sequences:* ***atga****, donor splice site predicted by Mutation tester,* ***ctgga****, donor splice site that occurred in vitro.* (**B**) **MaxEntScan of the possible 5’ (donor) and 3’ (acceptor) splice sites.** Since the ***ctgga*** sequence is repeated before (exon 49) and after (exon 62) the junction, we hypothesized two possible 5’ splice donors and two 3’ splice acceptors. For all, we calculated the MaxEnt score; according to the maximum entropy model, the most probable donor/acceptor splice sites are the second ones (seqq >2 and >4).

## Supplementary Figure S3


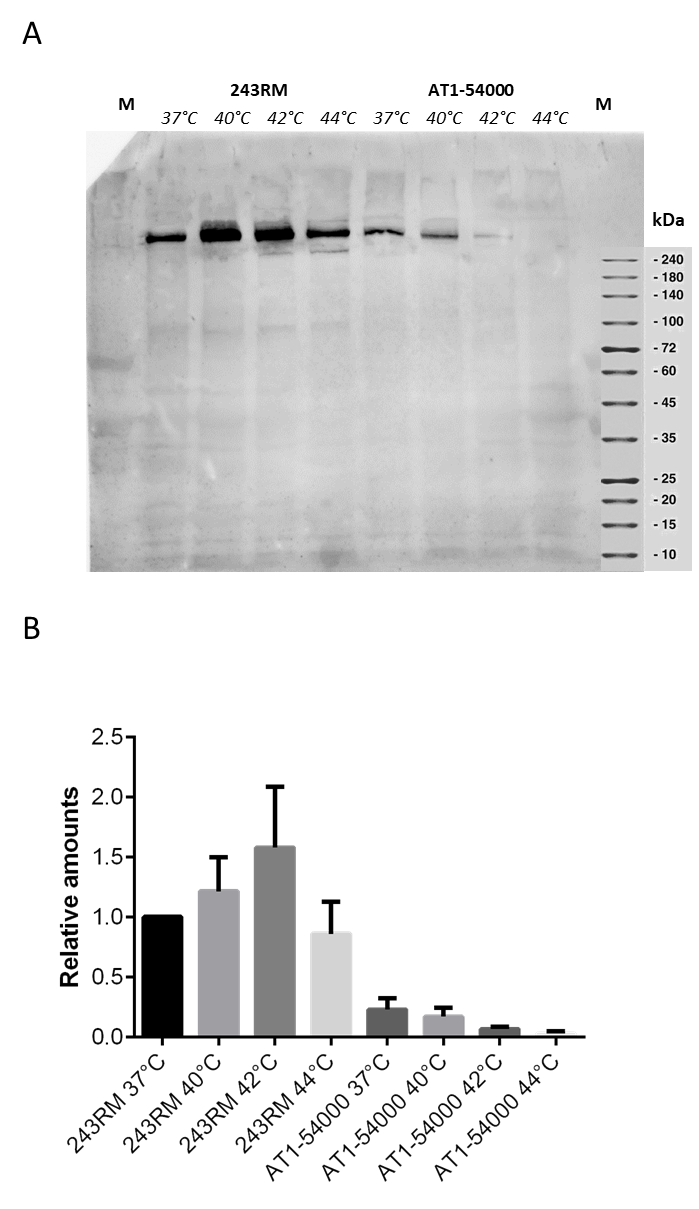


**Figure S3. Protein stability assay.** (**A**) Western blot analysis of ATM protein in WT and AT1-54000 cells after 1h at 37°, 40°, 42° or 44°C. (**B**) Quantification of the relative amounts of ATM protein in the total cell extracts of WT and AT cells shown in A. Blots shown are representative and values are the means and SEM of four independent experiments.

## Supplementary Figure S4


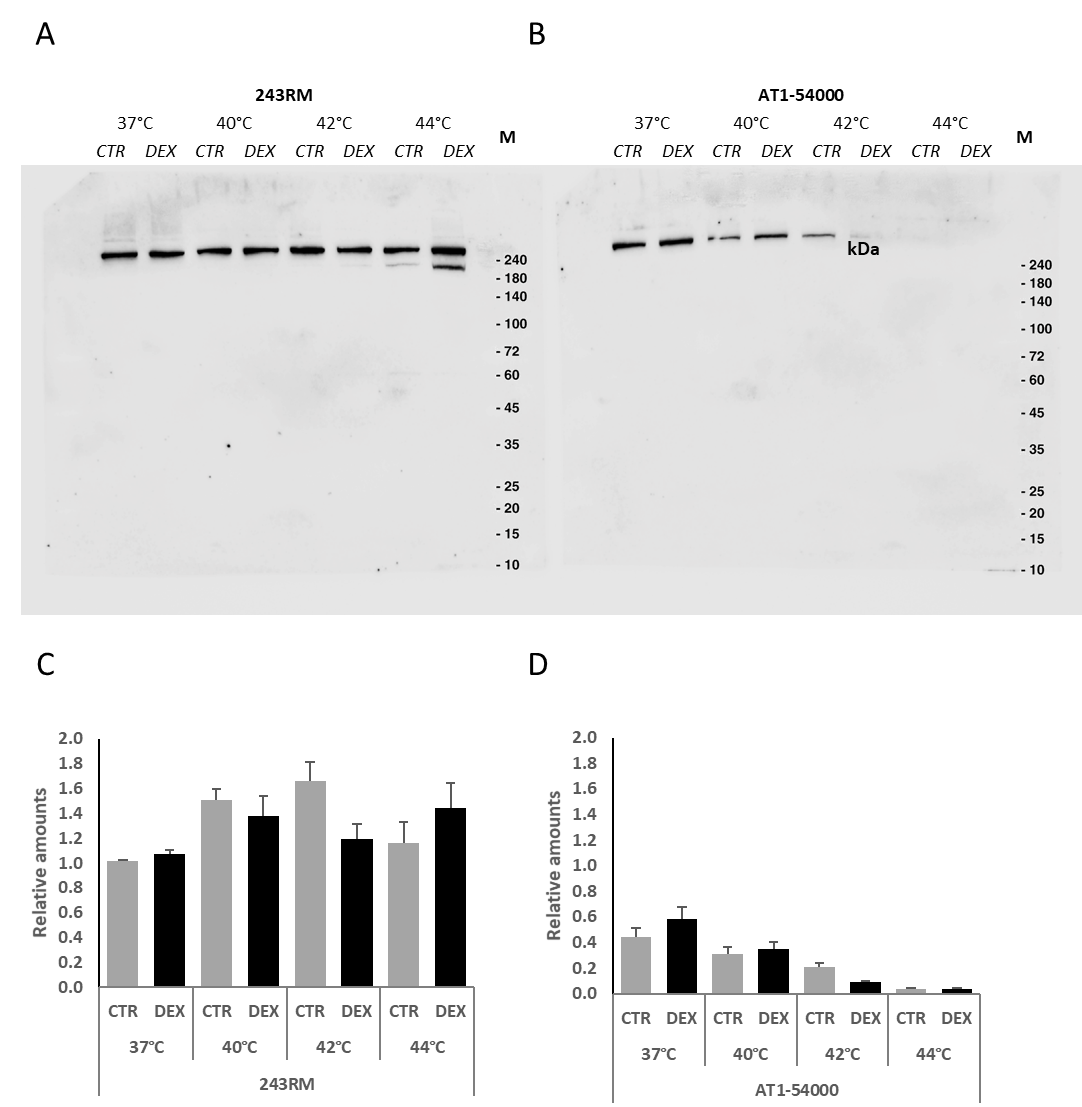


**Figure S4. Effect of DEX on protein stability.** (**A,B**) Western blot analysis of ATM protein in WT and AT1-54000 cells, treated or not with 100 nM DEX for 24h, after 1h at 37°, 40°, 42° or 44°C. (**C,D**) Quantification of the relative amounts of ATM protein in the total cell extracts of WT and AT cells shown in A and B. Blots shown are representative and values are the means and SEM of four independent experiments.

## Supplementary Figure S5

Healthy donors

AT heterozygous

carriers

Typical AT

patients

**Supplementary Figure S5. p53-MCL in AT1-54000 and AT1-54001 LCLs**. Bar graph representing the percentages of p53-MCL in AT1-54000 and AT1-54001 LCLs in untreated (Ctrl) and after DEX treatment.
